# Neuropathic Pain Prevalence of Older Adults in an Urban Area of Iran: A Population-Based Study

**DOI:** 10.1155/2019/9015695

**Published:** 2019-01-02

**Authors:** Reza Salman Roghani, Ahmad Delbari, Mohsen Asadi-Lari, Vahid Rashedi, Johan Lökk

**Affiliations:** ^1^Department of Neurobiology, Care Sciences and Society, Division of Clinical Geriatrics, Karolinska Institute, Stockholm, Sweden; ^2^Physical Medicine and Rehabilitation Research Center, Shahid Beheshti University of Medical Sciences, Tehran, Iran; ^3^Iranian Research Center on Aging, University of Social Welfare and Rehabilitation Sciences, Tehran, Iran; ^4^Department of Epidemiology, School of Public Health, Oncopathology Research Center, Iran University of Medical Sciences, Tehran, Iran; ^5^School of Behavioral Sciences and Mental Health (Tehran Institute of Psychiatry), Iran University of Medical Sciences, Tehran, Iran

## Abstract

**Background:**

Pain prevalence would increase as the population grows older, but the exact prevalence rate is not apparent in Iran.

**Objectives:**

This study, therefore, set out to reveal the prevalence of pain, especially neuropathic type and explore its associated comorbidities among Iranian older adults in a large urban population-based survey.

**Methods:**

5326 older people, aged ≥ 60 years, were randomly chosen by a multistage, cluster sampling method. The selected people then were interviewed by using the following instruments: a standard questionnaire about pain, questions of interview part of Douleur Neuropathique 4 Questions (DN4) and its comorbidities, GHQ-28, and a sociodemographic checklist. Descriptive statistics and multiple regression analysis were conducted to analyze the gathered data.

**Results:**

The average of the participants' age was 68.92 ± 7.02 years. Of 5326 participants, 2529 (47.5%) of participants were male. About one-third of this population had chronic pain. Chronic neuropathic pain prevalence was 13.7% and nociceptive in 30%. Knee pain (20.6%) and feet dysesthesia (7.8%) were the most common sites of nociceptive and neuropathic pain, respectively. Results of multiple regression analysis revealed that the major comorbidities of chronic pain were osteoporosis, disability, diabetes mellitus, and stroke. Neuropathic pain experiences were significantly associated with GHQ-28 scores (t=-11.42, P<0.001).

**Conclusions:**

In addition to neuropathic pain, other subtypes of pain prevalence and the comorbidities are determined in the community-dwelling elder adults. This study highlights the importance of neuropathic pain and its adverse consequences and can be used to manage this populations' needs in Iran effectively.

## 1. Background

International pain association has defined the pain as an experience, sensory, and emotional, that is related to damage, actual, or potential [1]. Pain can be classified according to its pathophysiology into nociceptive pain, neuropathic pain, and pain without a known somatic background [2]. Neuropathic pain is a result of damage to the nervous system, but the nociceptive pain is a result of injury to nonneural tissues when nociceptors are activated [3]. In population-based studies, neuropathic pain has been more severe than other types of pain [4–6], and it is important to identify patients with neuropathic pain and neuropathic components because conventional analgesic treatment may be less effective in older adults [7, 8]. Some scientists believe that pain is a subjective experience [9]. Therefore, personal reports of pain can act as a channel to ease the information sharing between professionals and patients [10]. Older people are a specific population for researchers and clinicians who deal with pain; as their prognosis is often less favorable, their independence is gradually lost and too much comorbidities [11, 12].

There are some studies with complaints of daily pain by half of the older participants [13]. According to a plethora of studies that tried to reveal the effects of pain on functioning aspects, the prevalence of functional limitations among older adults ranges from 10 to 61% [14–17]. Such a variability in frequencies may be because of methodological differences, participants' features, and cultural specificities [18, 19]. The International Classification of Functioning (ICF) model gives us a framework that can be used to recognize normal functioning from abnormalities [20]. According to this model, functioning at individual and societal levels can be estimated by activities and participation domains, respectively. In clinics, healthcare professionals should measure the aspects of functioning covered by these two domains; these measures can help in better management and alleviation of pain and its comorbidities [21].

Multiple comorbidities serve as hallmarks in geriatrics. Estimates of comorbidities prevalence may differ depending on the target population and the measures used, but most of the older people suffer from, on average, four or more comorbid health problems [22, 23]. In fact, common old age disorders like cardiovascular diseases, stroke, diabetes mellitus, obesity, cognitive impairment, anxiety, and depression are associated with pain. Also, the relationship between pain and disability might be related to its coexisting comorbidities or a consequence of pain [24–28].

The population of older people is growing globally, faster than the growing of population as a whole [29]. According to some estimations, the proportion of individuals aged > 60 years in Iran will have increased from 8.2 to 20% by 2020 [30]. Current literature about pain and comorbidities among Iranian older adults lacks consistency regarding definitions [31–33]. A wide and loosely specified array of variable are used to define pain and comorbidities [34]; this matter makes it difficult to compare the results. The current research contributes to knowledge by addressing two significant issues, determining the prevalence of chronic pain (neuropathic and nociceptive) in specific parts of body and its comorbidities in elder population of an urban area of Iran.

## 2. Methods

### 2.1. Design and Participants

Pooled data was derived from the second round of a large population-based survey, which was conducted in 22 districts of Tehran, the capital of Iran, in 2013-14. Face-to-face interviews were conducted in participants' houses by trained interviewers to gather the required data [35].

### 2.2. Sampling

To select the participants, we used a multistage random sampling method as follows. First, the city districts were taken as strata. Second, 200 clusters were randomly chosen in each zone. Finally, eight households were selected in each cluster using a systematic random sampling method. All household members were treated as primary sampling units. Using Cochrane formula, the sample size in each district was estimated to be 1535 families. However, to control for sampling errors and reach to higher precision, the sample number was increased to 1600 households in each district, regardless of their population size. The sample size, in total, was 34116 households comprised of 118542 individuals from 22 districts and 368 neighborhoods [36]. A total of 5326 individuals aged ≥ 60 years were included into the study. Other details of the sampling process are covered in our previous work [37].

### 2.3. Instruments

A sociodemographic checklist including questions about age, gender, marital status, education, occupation, weight, and height was completed by the participants. Mental wellbeing of the participants was assessed by General Health Questionnaire-28 (GHQ-28). The GHQ-28 was developed to assess psychosocial functioning of people and has been validated by clinical assessments [38]. GHQ-28 is a valid and reliable psychiatric screening tool among Iranian older adults [39].

All the participants were interviewed using a specific questionnaire about pain (present, last year, chronic pain, and pain-related appointment with a physician) and their comorbidities. The questions and the definition of disability are described elsewhere [37]. Various types of pain were defined as follows: chronic pain (a pain that lasts for more than three months), neuropathic pain (questions of interview part of Douleur Neuropathique 4 (DN4) pain characteristics: burning, painful cold, electric shock, and associated symptoms: tingling, pins and needles, numbness, and itching), and nociceptive pain (pain originated in a specific part of body without above-mentioned characteristics). The DN4 questionnaire was originally developed and validated in French by Bouhassira et al. in 2005, which is one of the most widely used questionnaires [40]. It is a clinician-administered questionnaire consisting of 10 items. Seven items related to pain quality (i.e., sensory and pain descriptors) are based on an interview with the patient, and 3 items based on the clinical examination are related to the presence or absence of touch or pinprick hypoesthesia and tactile allodynia [41]. Experts assessed the questionnaire, and its face and content validity were proven by a panel of national experts from various disciplines [42] also, the same Persian version of interview part of DN4 used for validation and reliability later [43].

### 2.4. Data Analysis

Data obtained from the questionnaires were initially analyzed by descriptive statistics. Data analysis was conducted using SPSS-20 (Armonk, NY: IBM Corp.). Independent t-test, chi-square test, and multiple regression (Enter method) were used to test associations between the variables.

## 3. Results

A total of 5326 older adults participated in the study. The mean of participants' age was 68.92 ± 7.02 years (Range: 60-90, SE: 0.096). Sociodemographic features of the sample are presented in Table 1.

Table 2 demonstrates the prevalence of pain by location in the study sample.

The findings revealed that 70.8% of participants reported pain and 31.7% had chronic pain in one specific part of the body. The rates of chronic neuropathic and nociceptive pain prevalence were 13.7% and 30%, respectively. Results of multiple regression revealed that the significant comorbidities of chronic pain (neuropathic and nociceptive) were osteoporosis, disability, diabetes mellitus, and stroke (Table 3). The findings highlighted that the 86% of older adults had appointments with a physician for their pain.

There is a significant statistical difference between male and female regarding chronic pain experiences, both neuropathic (P<0.001, X2= 45.525) and nociceptive (P<0.001, X2= 61.178), which means that female is more affected than male.

According to the findings, there was no significant relationship between pain and sociodemographic characteristics (i.e., age, marital status, education, occupation, and Body Mass Index (BMI)).

In terms of GHQ-28 scores, the authors found a significant difference between those participants with and without pain, both neuropathic and nociceptive (Table 4).

## 4. Discussion

This study aimed to estimate the prevalence of neuropathic pain and explore its associated comorbidities among Iranian older adults in a large population-based survey. Pain is a day to day challenge for older adults and they are more prone to pain because of different age-related problems and diseases. Chronic neuropathic pain prevalence in our study was 13.7%, a little bit higher than similar studies. Population surveys in France [5], Morocco [6], and UK [4] and a recent systematic review of epidemiological studies [44] reported 6-10% prevalence for neuropathic pain. It is noteworthy that older adults with neuropathic pain use more health care compared with patients with nociceptive pain [45]. Argoff argued that the burden of neuropathic pain in older adults may be uniquely experienced for several reasons. First, older adults have the potential for greater pharmacokinetic and pharmacodynamics challenges compared to younger individuals, due to the former's increased likelihood of comorbidities and need for multiple medications. Second, cognitive dysfunction is more common in older patients compared with younger individuals, and may cause further challenges for clinicians and other care providers who are involved in assessing pain and responses to therapy. Finally, various other social factors, including lack of access to medical treatment, may result in the undertreatment of pain within older adults [46]. It should be emphasized that prevalence of neuropathic pain is driven based on question part of DN4 and it may be underestimated, and this is one of our limitations.

In our study, more than two-thirds of participants reported pain in overall and about one-third had chronic pain. However, a plethora of research has revealed that chronic pain prevalence among elderly populations varies considerably, ranging from 27% to 53% [47–50]. Inconsistency in pain prevalence in different studies has been due to various factors like pain definitions and recall periods [51].

The present finding also supports previous studies which concluded that the most common pain was in the back and knee parts. In older adults, benign or mild back pain seems to be less frequent compared with other age groups, but they experience more episodes with severe or disabling back pain [52]. Back and knee pain proved to be significant contributors to individuals' quality of life (QoL), and proper management of these conditions can help improve QoL [53, 54].

Our finding indicated that women were more affected by pain. This finding is supported by both cross-sectional [48, 55], review studies conducted by Bartley & Fillingim [56] and Fillingim et al. [57], and a cross-national study among 61,157 older adults in Europe [58]. There is still no proven evidence to justify the etiology of sex differences in terms of pain. However, many studies support differences in perception of pain due to biological, psychosocial, and cultural differences [56, 57, 59], but also gender differences in roles, beliefs, or coping strategies could contribute to the observed differences between men and women [60]. Other findings illustrated that there is no significant relationship between pain and sociodemographic characteristics, which was inconsistent with Lekpa et al. [61] and Pazzaglia et al. study results in sub-Saharan African elderly [62].

Similar to prior research, it was found that pain was associated with comorbidities and disabilities. In Concord Health and Ageing in Men Project (CHAMP), a cohort study among men aged 70 years and over in Australia, Blyth et al. observed that pain, frailty, and comorbidity can cooccur [63]. Also, Leong et al. reported an association between high comorbidity and pain severity among pain clinic attendants [64]. Applying a new measure for comorbidity and disability definition, this finding was confirmed in a current study with a larger sample of participants. Neither for comorbidities and chronic nor for mental health we could not establish a cause and effect relation in this study. Large community-based and cluster sampling is one of this study advantages and participants were not selected based on pain-related help-seeking behavior.

Less is known about the psychological dysfunction associated with pain among older adults [65], and the association between variability in pain experience and mental health is bidirectional [66, 67]. We found significant mental health difference between groups consistent with findings of past studies by López-López et al. in Spain [68], Dragioti et al. in Sweden [69], and de Koning et al. in an analysis across 6 European cohorts [70]. However, this difference could be due to pain or other comorbidities. In 2018 Brooks et al. demonstrate that pain and depressive symptoms may be linked to functional limitations [71], also Freynhagen et al. confirmed that depression and anxiety appear to be more common in patients with neuropathic pain compared to those without [72]. Based on the second wave of Healthcare for Communities (HCC2), Brennan Braden et al. discovered that chronic pain leads to more use of mental wellbeing services and higher distress in healthcare settings among older adults [73].

## 5. Conclusion

In addition to neuropathic pain, other subtypes of pain prevalence and the comorbidities are determined in the community-dwelling elder adults. This study highlights the importance of neuropathic pain and its adverse consequences and can be used to manage this populations' needs in Iran effectively.

## Figures and Tables

**Table 1:** Sociodemographic characteristics of the sample.

**Variable**		**N = 5326**	
		**n**	%
Gender	Male	2529	47.5
	Female	2797	52.5

Age	60-69	3070	57.6
	70-79	1785	33.5
	80-89	453	8.5
	≥ 90	18	0.3

Marital Status	Single	65	1.2
	Married	3811	71.6
	Divorced	83	1.6
	Widow	1367	25.7

Education	Illiterate	1388	26.1
	Elementary	1134	21.3
	Guidance school	808	15.2
	High school	397	7.5
	Diploma	727	13.7
	Academic	827	16.4

Occupation	Employed	595	11.2
	Housekeeper	1893	35.5
	Retired	2685	50.4
	Unemployed	153	2.9

BMI	< 20	97	1.8
	20-25	2263	42.5
	25-30	2087	39.2
	> 30	879	16.5

**Table 2:** Prevalence of pain by location among two groups of older adults (N=5326).

**Variable**			**n**	%
Acute	Neuropathic	Hand	437	8.2
		Feet	519	9.8
		Radicular neck pain	261	4.9
		Radicular back pain	420	7.9
		Overall	879	16.5
	Nociceptive	Back	1171	22
		Neck	522	9.8
		Shoulder	694	13.1
		Knee	1638	30.8
		Overall	2040	38.3
	Overall		2082	39.1

Chronic	Neuropathic	Hand	314	5.9
		Feet	416	7.8
		Radicular neck pain	172	3.2
		Radicular back pain	291	5.5
		Overall	731	13.7
	Nociceptive	Back	727	13.6
		Neck	304	5.7
		Shoulder	471	8.8
		Knee	1098	20.6
		Overall	1598	30.0
	Overall		1688	31.7

Overall Pain (Acute + Chronic)			3770	70.8

**Table 3:** Multiple regression analysis for the association of chronic pain and its comorbidities.

**Model**	**B**		**Beta**		**t**		***P-*value**		**R**	
	Neu	Noc	Neu	Noc	Neu	Noc	Neu	Noc	Neu	Noc
(Constant)	0.263	0.263			7.782	7.576	< 0.001	< 0.001	0.161	0.160
Osteoporosis	0.104	0.094	0.092	0.081	6.403	5.632	< 0.001	< 0.001		
Disability	- 0.070	- 0.071	- 0.060	- 0.062	- 4.101	- 4.571	< 0.001	< 0.001		
Gender	0.051	0.069	0.060	0.079	4.198	5.510	< 0.001	< 0.001		
Diabetes mellitus	0.061	0.052	0.052	0.043	3.805	3.133	< 0.001	0.002		
Stroke	0.115	0.093	0.032	0.029	2.317	1.932	0.021	0.047		

^*∗*^Neu: neuropathic. ^*∗∗*^Noc**: **nociceptive.

**Table 4:** Mean differences of GHQ-28 score based on neuropathic and nociceptive pain experiences.

**Variable**	**Neuropathic**		**t**	**df**	***P*-value**	**Nociceptive**		**t**	**df**	***P*-value**
	**No**	**Yes**				**No**	**Yes**			
	M ± SD	M ± SD				M ± SD	M ± SD			
GHQ-28	50.12 ± 10.69	54.07 ± 11.17	- 11.42	5324	< 0.001	50.07 ± 1072	53.89 ± 11.07	- 11.36	5324	< 0.001

## Data Availability

The data used to support the findings of this study are available from the corresponding author upon request.
